# Blob-ology and biology of cryo-EM: an interview with Helen Saibil

**DOI:** 10.1186/s12915-017-0417-z

**Published:** 2017-08-31

**Authors:** Helen R. Saibil

**Affiliations:** Department of Biological Sciences, Institute of Structural and Molecular Biology, Birkbeck College, London, UK

## Abstract

Helen Saibil is Bernal Professor of Structural Biology at Birkbeck, University of London. After undergraduate work at McGill University, Canada, Helen moved to London for her PhD at Kings College. After stints at CEA Grenoble and the University of Oxford, she moved to Birkbeck where her lab studies the operation of macromolecular machinery—including molecular chaperones, protein folding/misfolding, and host cell interactions with pathogens. Helen is a Fellow of the Royal Society, Fellow of the Academy of Medical Sciences, and an Honorary Member of both the British Biophysical Society and the Royal Microscopical Society. She talked to us about the background, recent developments, and future prospects in cryo-electron microscopy.

## Cryo-electron microscopy is becoming, or has already become, a mainstream technique for studying biological structure. Can you give a background of how we’ve got to where we are now—the history of the technique?

**Fig. 1.**
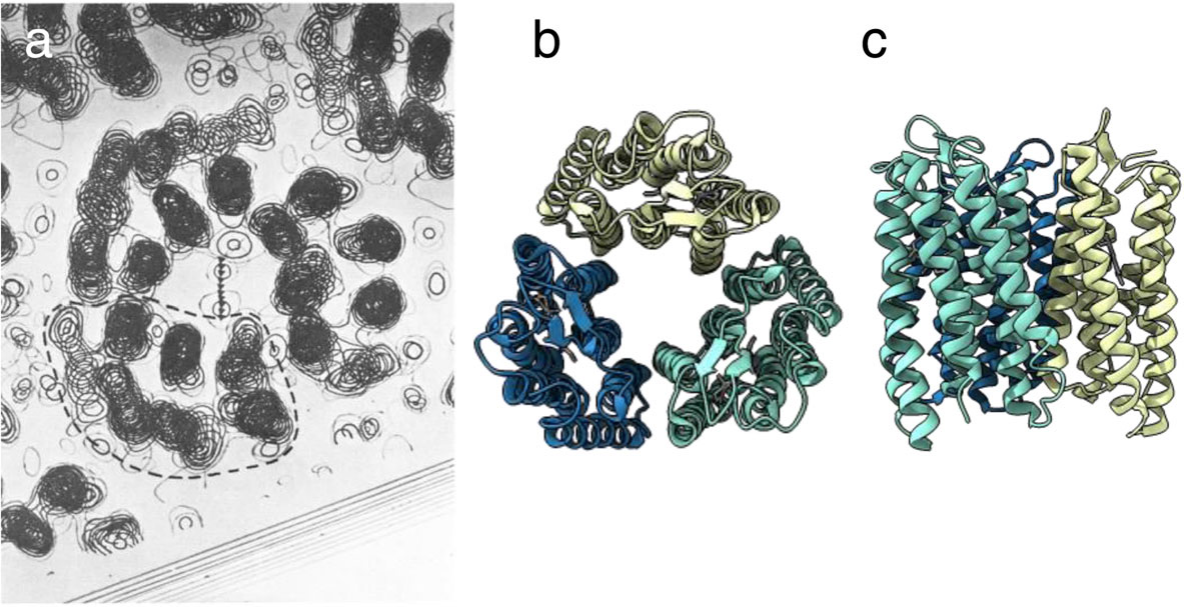
Structure of bacteriorhodopsin. **a** Part of the 3D potential map of bacteriorhodopsin as described by Henderson and Unwin [1]. **b, c** ‘Top down’ (**b**) and transmembrane (**c**) structure of bacteriorhodopsin taken from PDB (PDB ID 1FBB). ‘Blobs’ in **a** are helices viewed from above, as in **b**. Image in **a** reprinted by permission from Macmillan Publishers Ltd: Nature [1], copyright 1975

I didn’t start at day zero, but I was a graduate student in the 1970s at King’s College (London) where diffraction methods and EM were being used to study biological assemblies, so I was aware of the structural techniques being used. The big excitement during my PhD was when Richard Henderson and Nigel Unwin came up with the structure of bacteriorhodopsin by electron diffraction and imaging (Fig. 1) [1]. The images were blank, because the contrast of individual unit cells was too weak to see, but the diffraction patterns calculated from images of the arrays of protein in the membrane showed many, sharp diffraction spots: that was really revolutionary. The idea of getting 3D structures from electron microscopy images had already been worked out—collecting images of the assembly from different view orientations to get the three-dimensional (3D) density. That was very much in parallel with medical tomography, because the principles are exactly the same—getting projections—like chest X-rays—from all angles around the patient. The bacteriorhodopsin work yielded the very first view of the alpha-helices of a membrane protein going through the membrane. Because these were well ordered two-dimensional crystals, and bacteriorhodopsin arrays in the membrane happen to make a material that's like concrete, it didn't fall apart in the high vacuum of the electron microscope column. Almost no other biological sample would resist a high vacuum. So they chose an ideal object to develop the methods. Seeing a protein structure emerging from apparently blank images was absolutely magic.

The diffraction approach allowed them to use the data just from the spots—throwing away all the noise not at lattice positions, giving a vast improvement in signal to noise ratio, and thus revealing the alpha-helices in the projection. This spectacular result sparked my interest in electron microscopy. That was the start of getting molecular structures of large assemblies: it resolved alpha-helical secondary structures, enough to start understanding the organization of biological molecules in 3D.

At that time this approach was feasible for ordered arrays, well-ordered helices or icosahedral viruses, assemblies with symmetrically arranged units. Methods for structure determination of asymmetric or irregular objects, which include most of the interesting and important machines in biology, were slowly but steadily developing elsewhere, notably in the lab of Joachim Frank in the US.

## Where did the ‘cryo’ come in?

Another important development that was happening gradually through the ‘70s and ‘80s was to stabilise the sample by rapid freezing, to avoid the damaging dehydration. Conventionally, biological samples in EM are dehydrated and stained, so that what you're looking at is uranium or some other heavy metal rather than the biological molecules. Bob Glaeser and Ken Taylor in the States, and Jacques Dubochet in Switzerland and Germany, started to figure out ways of capturing biological samples in their native, hydrated state by rapid freezing. With appropriate equipment, the frozen samples could remain stable in the high vacuum of the microscope.

Jacques Dubochet got vitrification (turning the water in the aqueous sample into a solid, glass like state) to work in a simple and elegant way by just plunging thin layers of molecules, complexes, viruses, membranes or even thin cellular structures into a cryogen that would remain liquid while rapidly transferring heat from the specimen [2]. This approach really took off, because you could do EM of real biological samples, isolated and purified, or of thin regions of cells. The technology for keeping the samples cold and stable in the microscope has steadily improved over the decades. At the same time, the software developments were needed in order to deal with the low contrast and low signal to noise ratio in images of unstained biological samples.

## Did people catch on quickly to the idea?

At that time, many of the crystallographers thought electron microscopy was just blobology. Most of them were pretty sceptical about this low-resolution imaging. I remember an incident in Oxford when I was at the graphics terminal with some image on the screen, and a crystallographer came up behind me saying, pityingly, “oh, has it gone out of focus?”

But a few people—even some crystallographers—realized that EM was going somewhere, and it steadily carried on improving, even though few others noticed. One of the big inspirations for development of single particle analysis, the study of individual molecules or complexes in solution, has been the structure of the ribosome. Ribosome structure determination has driven forward many of the software methods [3, 4].

## There was a revolution of sorts fairly recently with direct electron detectors. How did the field progress up to and around then?

The resolution crept up gradually over the years, as the hardware improved. Cryo-microscope stages got more stable, field emission gun sources were developed, giving more coherent illumination, better contrast and resolution. But for a long time, the images were recorded on photographic film, which was still in common use within the last decade. Until 2013 the only electronic detectors for EM were CCDs—charge-coupled devices. Although much more convenient than film, they require that the electrons are converted into visible light for detection, degrading the signal by scattering and making it very difficult to recover high resolution information.

Just as introduction of vitrification in the 1980s was a huge leap forward for biological imaging, the development of direct electron detectors led to a gigantic jump forward from 2013. These detectors respond directly to the electron signal, are much more sensitive and much faster than CCDs. The sensitivity is a major issue, since the resolution is limited by electron beam damage—the fewer electrons needed to record an image, the better preserved the structure. In addition, the high speed of the new detectors gave an unexpected benefit: Previously, the electrons would hit the ice, causing the sample to move during the exposure, so that fine details would be blurred and irretrievably lost. Because the direct detectors are so fast, they can record movies instead of single images, with enough signal in each frame of the movie so that the frames can be aligned with each other to recover signal that was lost to motion blur. This restoration of lost data was magic in the same way that extracting the bacteriorhodopsin structure from noise was magic.

The resulting vast improvement in cryo-EM data quality really kicked off the revolution: the software works much better, because the signal is so much better, giving a positive feedback because many structures started going to high resolution. A particularly effective way of using these detectors is electron counting. The idea is very similar to super resolution optical microscopy methods such as PALM or STORM, in which sparse events can be accurately localised by finding the peak position of the blob of intensity created by arrival of a single photon. The same principle is used in electron counting—if the rate of electron arrival is low enough, the position of each individual peak can be accurately determined and scaled to a count of 1, which greatly improves the accuracy and resolution of the image data.

## And the benefits went beyond ‘just’ increasing the resolution?

What was perhaps counterintuitive was that, in addition to preserving the high-resolution signal much better than before, electron counting also greatly improves the low-resolution signal. One of the properties of cryo-EM is its poor contrast at low resolution, making it very hard to see small features. So if you have a small particle, you may not be able to find it or determine its orientation. Improved low-resolution contrast makes it possible to detect and process small particles, and generally facilitates particle alignment. The improvements in low as well as high resolution really made things fly. The gains have been amazing for those in the field, and single particle cryo-EM has now really taken its place next to protein crystallography as a mainstream structural biology method.

A unique and incredibly interesting feature of the single particle approach is the ability to sort out structural heterogeneity. This is another aspect that has been steadily developing over the decades. A large data set of particles will contain different projections of the structure seen from different orientations, and the main task in 3D reconstruction is to determine their angular relationships. But if there are other variations, such as the presence or absence of an extra element like a ligand—say an initiation factor on the ribosome—you can statistically classify and distinguish the particles containing the ligand from the ones without it. Often, the most interesting features of a biological machine are its conformational fluctuations. The moving parts are the ones carrying out the machine’s function.

## So when we think of structure, it’s really dynamic rather than a single, fixed structure?

Macromolecules have dynamic fluctuations that can be trapped by vitrification but can’t be biochemically separated. With a large, high quality single particle image data set, the variations can be statistically analysed, for example by principal component analysis, similar to the analysis of multiple sequences in bioinformatics, or the closely related approaches of multivariate statistical analysis or eigenvector analysis. These methods can find the principal variations in the data set, and sort particles according to their conformational variations.

**Fig. 2.**
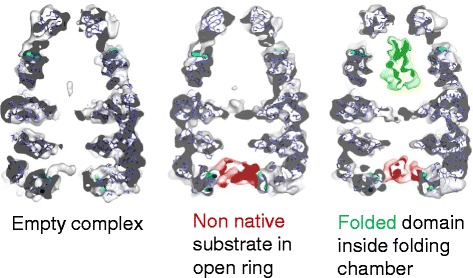
Structures of chaperonin complexes with folding intermediates of a bacteriophage capsid protein. Sections through reconstructions of the complexes were sorted into three classes. One class did not show substrate density, and two others had non-native protein (*red*) bound in the open ring. One of these bound classes also had density inside the enclosed folding chamber, with the shape of the folded large domain of the gp23 substrate protein (*green*). Image adapted from [5]

In our work, we used statistical analysis to identify chaperone complexes with a particular non-native protein bound inside them and those without the bound protein, and to find different conformations of the non-native proteins: things that are intrinsically very heterogeneous [5] (Fig. 2). Many labs work on ribosome complexes, which have moving parts and contain different binding factors—lots of variations have been found by sorting image data sets into multiple structures.

Image classification is not a true single molecule approach, but it's pseudo single molecule, because it can sort a large data set—many images—into subsets on a statistical basis. With enough images in each subset, it is possible to determine the corresponding set of 3D structures. Single particle classification is a very powerful tool—almost every other structural method averages all the molecules in the sample, blurring out the variable parts.

## And the more and better data you're generating from the microscopes themselves requires the data analysis to co-evolve?

**Fig. 3.**
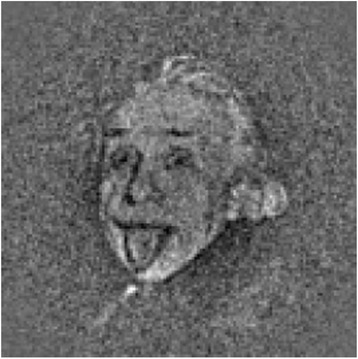
Einstein from noise. An image of Einstein appears from 1000 images of pure white noise by using a normalized cross-correlation function and the photo as a model. Reprinted from [6], *Journal of Structural Biology*, vol. 166, M. Shatsky et al., A method for the alignment of heterogeneous macromolecules from electron microscopy, pp. 67–78, Copyright 2009, with permission from Elsevier

Yes. The software is getting more automated, and the results are improving because the images are better. The methods for image processing and analysis of the resulting maps are still evolving. One of the big problems used to be the starting model. If you were working on a new, unknown structure, you could get it wrong by using an incorrect starting model with a noisy and/or heterogeneous data set. This phenomenon has been likened to Einstein from noise: if you have a large data set of random noise and you align it to a picture of Einstein, the noise features will align to create the image of Einstein (Fig. 3) [6]. That's less of a problem now because the data quality is better—and algorithms are now available that search broadly to automatically find the correct starting model.

## You mentioned that looking at real biological material, in situ, is a key aim—so what are the current advances—or challenges—of newer developments, like electron tomography?

Structural analysis of cells and tissues is not as mature. I think the real frontier now is understanding biological machinery in situ—in the cell, in the tissue—and that's much harder. The electrons don't penetrate through regions thicker than the thinnest edge of adherent cells, excluding the study of most cellular structures. It is therefore necessary to cut sections somehow if you want to see through the nucleus or adjacent structures, or for any tissue sample. This is a difficult task: the most accessible approach is to slice through high-pressure frozen samples with a diamond knife in a cryo microtome, but mechanical distortions compress the section and limit its thickness to only 50–80 nm, which is too thin to capture most cellular structures.

## So you need to improve this sectioning

**Fig. 4.**
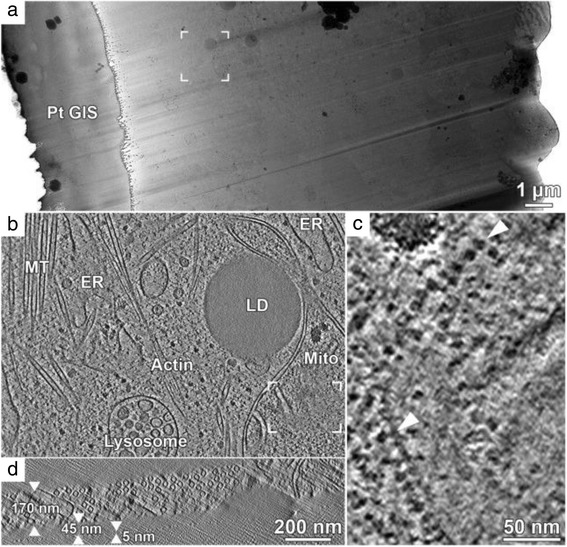
Cryo-electron tomography on a FIB lamella of a HeLa cell. **a** 2D transmission electron microscopy montage of a HeLa cell FIB lamella. Left: lamella top with an organometallic Pt layer. Right: thicker bottom side of the lamella. **b** x,y slice from a tomographic volume acquired at the framed area in **a**, showing a variety of organelles and cytoskeletal structures within the cytoplasm. MT: microtubules, ER: endoplasmic reticulum, LD: lipid droplet, mito: mitochondrion. **c** Enlarged area within the mitochondrion (framed region in **b** rotated by 90^o^). A row of ATP synthase complexes is visible along the cristae membranes in top view (*top arrowhead*) and in side view (bottom arrowhead). **d** Corresponding x,z slice of the tomographic volume in **b**. The lamella thickness is 170 nm with a 5 nm Pt surface coating sputtered after lamella preparation. An additional ~45 nm layer of water vapor condensed on the finished lamella over the course of an hour during preparation. Tilt-series was recorded with the Volta phase plate, a target defocus of 0 μm and an object pixel size of 0.421 nm. Reprinted from [7], *Journal of Structural Biology*, vol. 197, M. Schaffer et al., Optimized cryo-focused ion beam sample preparation aimed at in situ structural studies of membrane proteins, pp. 73-82, Copyright 2017, with permission from Elsevier

There is currently no solution for the mechanical problems with sectioning vitrified samples. But there's an alternative method called cryo-focussed ion beam milling in which the excess material is burned off with a gallium ion beam in a cryo scanning electron microscope. Layers of material are removed to leave a thin lamella 100–300 nm thick, and the sample is then transferred into the transmission EM for tomography. This method gives excellent results but it is extremely slow, and there are not yet many working systems running (Fig. 4) [7].

## Presumably having a slab of tissue comes with its own challenges!

Tomography can be done on any biological sample of suitable thickness, since it doesn't require averaging of many different views of the object. The cost of this broad applicability is that it needs a whole series of views of the area of interest recorded at different tilt angles. There are two problems with recording a tilt series: the electron dose, and hence radiation damage, accumulate during multiple exposures of the same area. In addition, the sample cannot be tilted more than about 70 degrees because the path of the beam through the sample becomes too long. So there is always missing data: the raw tomogram doesn't contain all the information, with very poor resolution in the beam direction. For biological samples, the accumulated electron dose imposes an ultimate limit on resolution, since they are very beam sensitive. That's not true in materials science—if you’re looking at a gold lattice you can see columns of gold atoms because you can use a high dose of electrons to record a clearer image. Nevertheless, despite their lower resolution tomograms can provide much unique information about the native structures of cells and tissues.

## It is possible to go further with repeating features in tomograms. How does that work?

**Fig. 5.**
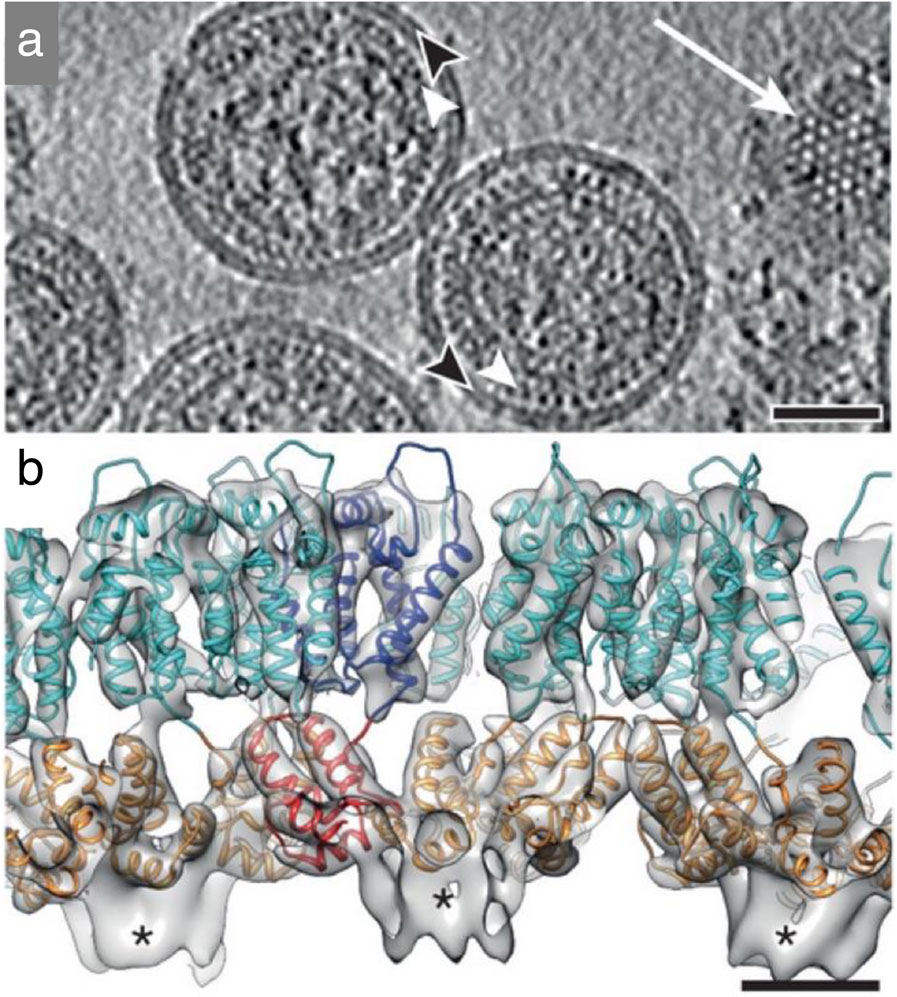
Structure of the immature HIV-1 capsid. **a** Computational slice through a Gaussian-filtered tomogram containing immature HIV-1 particles. *White arrowheads* indicate the immature capsid layer; *black arrowheads* indicate the membrane; *white arrow* marks a grazing slice through the capsid layer illustrating the hexagonal lattice. Scale bar, 50 nm. **b** Isosurface representation of the final structure showing the immature lattice in an orthogonal view. Isosurface threshold value is 2*σ* away from the mean. High-resolution structures for capsid N- (*cyan*) and C-termini (*orange*) have been fitted into the density. An individual capsid monomer is coloured *blue*/*red*. Unfilled densities marked with *asterisks* correspond to spacer peptides. Scale bar, 25 Å. Adapted by permission from Macmillan Publishers Ltd: Nature [8], copyright 2014

If you have repeating objects within a cell or tissue—like the surface spikes on an irregular virus, such as flu or HIV, you can use an image processing approach called sub-tomogram averaging to determine their structures (Fig. 5) [8]. The spikes in such viruses are irregularly arranged on a closed surface. They’re in all different orientations and when you reconstruct that virus in your tomogram, those spikes will be pointing in different directions. You can cut them out in little 3D cubes and then use those as a single particle dataset for classification and averaging.

This is more complicated than 2D single particle analysis—each cut out spike will have its own missing direction in the tilt data, which will distort the structure in a different way, and you have to take account of that. But if you have randomly oriented objects in your tomogram, when you put them all together the missing directions will fill in for each other, completing the data. Thus, sub-tomogram averaging both improves the signal to noise ratio of the structure by averaging, and fills in the missing directions from the different particles in the dataset. It’s slower and much more complicated, but gives you single particle analysis in three dimensions from many interesting biological structures.

A way to optimise tilt data collection for sub-tomogram averaging is to record the low tilts first, by starting the series at zero degrees and then swinging back and forth around zero to higher and higher positive and negative angles. The microscope stage must have good stability to operate this scheme, because it requires rotations through increasingly large angles [9]. The resulting tomogram has the best data at low tilt. The high dose is delivered for the tilt data, which is always of lower quality, because of the longer path length through the sample. The full tomogram is used to find the objects of interest in 3D, but when each object is used for averaging, it is possible to use only the lower tilt part of the data so that only the best data contribute to the average. With many particles distributed at different orientations the average can reach a very good resolution. This procedure is effectively like 2D single particle analysis on a 3D object. But the 3D information is essential to get to the right places first.

## How does cryo-EM fit together with and complement other microscopy techniques that are available?

A typical problem in electron tomography of a biological structure, especially if there’s only one of them in the cell, is to find it! And the particular slab that you have cut or milled must still contain it. This can be a challenging problem. The task consists of finding the thing you want in cryo-fluorescence, then transferring the sample to the transmission electron microscope, without ice contamination of the frozen sample, and then finding the right spot in the EM. There are also systems for combining various kinds of scanning EM with fluorescence built into the column.

This correlative microscopy approach requires fluorescence labelling. However, it is not possible to label cryo sections or lamellae with antibodies or other reagents, since they can't be thawed. So it is necessary to engineer a fluorescent tag on the gene of interest.

Continuing developments in correlative microscopy are very important for getting cell biology to the level of molecular complexes and assemblies. You need to know where you’re going, because cryo-tomograms of a cell are full of density features of unknown identity. Good signposts and careful experimental design are essential.

At present the equipment and facilities for doing this kind of work are not routinely available. But to understand molecular events in cell biology—the operation of biological machinery in situ—these approaches are indispensable.

## And how far does this interplay go? For example, doing correlative super resolution?

This is an exciting area. Cryo samples are in principle excellent for fluorescence because photo bleaching is practically eliminated at cryo temperatures. There’s one thorny problem—you have to immerse your high quality, expensive optical objective in liquid nitrogen or some other cryogen. They weren't designed with such treatment in mind, so this is a little tricky. The simple way to get around it is to have an air-gap, which limits the resolution. The high numerical aperture objectives need immersion, but you can’t have oil immersion in cryo. People are developing cryo super resolution by various tricks, either with gaps or by thinking about cryogenic liquids that can be used for immersion. Current systems use air-gap imaging, giving a limited but still useful form of super resolution.

## In practical terms, of course these machines are expensive and you need the infrastructure—how is that developing alongside the technological advances?

Unfortunately the machines are very complicated and prohibitively expensive! Time on high end microscopes is always in short supply. Especially for tomography you need a lot of microscope time. The UK is quite advanced in having a national facility for cryo-EM at the Diamond synchrotron. The field has trailed some 30 years behind X-ray crystallography but it is catching up quickly. It's much easier to do things the second time than the first time. So the field has copied what the crystallographers worked out over the decades, adapting existing systems and ideas for EM. In fact many crystallographers are now enthusiastically adopting electron microscopy and setting up new EM centres. This work needs some serious expertise and there's a huge demand for experienced people—the labs that train people have a pretty high employment rate of their alumni.

The single particle approach is working really well—the limit’s going to be the biochemistry, and understanding what you need to do to get your sample in the right state. The next step is in situ structures of cells and tissues. That is much slower. Eventually single particle work will get to the stage of X-ray crystallography where the users will press a button to record data and get their structure. But if there's anything complicated or it doesn't work, they won't know what to do and they’ll have to find the old guys!

## So how does this high-resolution microscopy and cryo-EM fit into the current drive for reproducibility in science—data sharing, for example; and is the field working in the same direction in this respect?

In single particle analysis this is fairly straightforward. The maps, the fits—and eventually the raw data—are deposited in public databases, and then other people can use or reanalyse it to make comparisons or get something new out of it. One thing that is not fully solved is determining a reliable measure of resolution for EM structures. There’s a generally used procedure for splitting the data in two independent halves, separately reconstructing and comparing them, and then determining at which resolution the correlation between the two half maps falls off—the resolution is determined at a defined correlation level. But this method is not always accurate and can be very misleading, not least because the resolution usually varies over the different parts of the structure. The lower resolution parts are often the interesting ones—the wobbly bits typically carry out the function. The agreement between two halves of the data set does not necessarily provide an absolute optical resolution from point to point.

There have been many arguments in the field about resolution measurement and whose method is better. This is important to the method developers. What matters to biologists is how reliable the structure is and what it tells you. Sometimes a low resolution map reveals more biological insight than a high resolution one.

There are still many challenges in the methods, but with good progress towards more automated, more routine, more robust cryo-EM methods, the focus is shifting to the biology. Once the problem is formulated, cryo-EM should provide the tools to do the right experiment. In this field, the biology's always the motivation: to find out ways of addressing biological problems, leading to new insights into how living things work.

Further reading: See recent review from Richard Henderson, “Overview and future of single particle electron cryomicroscopy” [10].
